# Hybrid Scheduling Model for Independent Grid Tasks

**DOI:** 10.1155/2015/692084

**Published:** 2015-10-12

**Authors:** J. Shanthini, T. Kalaikumaran, S. Karthik

**Affiliations:** SNS College of Technology, Coimbatore, Tamilnadu 641035, India

## Abstract

Grid computing facilitates the resource sharing through the administrative domains which are geographically distributed. Scheduling in a distributed heterogeneous environment is intrinsically very hard because of the heterogeneous nature of resource collection. Makespan and tardiness are two different measures of scheduling, and many of the previous researches concentrated much on reduction of makespan, which measures the machine utilization. In this paper, we propose a hybrid scheduling algorithm for scheduling independent grid tasks with the objective of reducing total weighted tardiness of grid tasks. Tardiness is to measure the due date performance, which has a direct impact on cost for executing the jobs. In this paper we propose BG_ATC algorithm which is a combination of best gap (BG) search and Apparent Tardiness Cost (ATC) indexing algorithm. Furthermore, we implemented these two algorithms in two different phases of the scheduling process. In addition to that, the comparison was made on results with various benchmark algorithms and the experimental results show that our algorithm outperforms the benchmark algorithms.

## 1. Introduction

Grid computing is defined as a coordinated resource sharing and problem solving dynamic, multi-institutional virtual organization [1]. The idea behind grid computing is apprehended and established by Ian Foster. Grid network spans across the administrative domains to complete the tedious mathematical calculations. The global grid forum [2] defines protocols for grid architecture that includes Grid Security Infrastructure (GSI), Monitoring and Discovery Service (MDS), Grid Resource Allocation Manager (GRAM), and Grid File Transfer Protocol (GFTP). The GSI is responsible for security issues in grid environment such as credential delegation and single sign-on. On each successful submission of a task, the GRAM initiates the MDS service to discover the suitable resources to execute the task. UNICORE presented in [3] allows secured and seamless access to distributed resources. The GRAM component decides which resource has to be allocated from the pool of suitable resources, which are collected by MDS. In [4] an Agent Based Resource Management System is presented which uses a prediction technique of PACE toolkit to predict the performance of applications running on local resources. An agent implementation presented here provides a service advertisement and discovery service for grid applications. In [5] novel iGrid architecture is designed to retrieve the resource information that gets updated frequently, whereas MDS does not support such frequent updates.

The grid scheduler makes the coherent and coordinated use of grid resources. The grid scheduler is more significant to grid environment where performance and quality of service are major concerns. The work of a grid scheduler is difficult and complicated compared to general distributed network scheduler. The GridWay [6] and Grid Service Broker [7] are some well-known grid schedulers. The grid scheduler has to work with different types of resource in which each of those schedulers follows a diverse local policy to access. Further, grid scheduler must work with local resource allocation managers (LRM), because the components have full control over the local resources. The grid schedulers are normally global schedulers by the classification [8]. The global schedulers use system related information which makes the scheduling well-organized and effective. The proposed system concentrates on schedule based approach [9], which supports many prospects of grid architecture. For actual scheduling process in a well-organized system or a schedule based system, a job needs start time and completion time of a particular task at the earliest. For each new instance of the scheduling, the planned system recomputes the schedule plan. The schedule based system is well suited for grid environments, and it supports resource reservation and planning. In contrast to the above-mentioned method, the queue based systems utilize the presently available resources for scheduling, which may result in poor system utilization and performance. FCFS, PBS, and condor are some renowned queue based systems.

## 2. Related Work

The role of the scheduler becomes vital in heterogeneous architecture; in addition to that performance, QoS and cost of computing are some of the primary concerns. There are many researchers who documented their efforts in efficiently solving the primary requirement. Opportunistic Load Balancing (OLB) [10] which is also called as myopic algorithm collects all incoming tasks and assigns them to machines in random fashion. XSuffrage [11] gives each task a priority value based on the suffrage, then tasks are scheduled based on suffrage value. DFPLTF [12] stands for Dynamic Fastest Processor to Largest Task First, as the name implies, it gives higher priority to larger tasks. Work Queue is another attempt; here the fastest processor gets more tasks than the slower ones. Min-min, Max-min, DFPLTF, and Work Queue (WQ) [13] are the representative algorithms in RR. In [14] round robin replication remote work queue (R3Q) algorithm is proposed, which combines the list scheduling task that replicate round robin techniques.

RR–TPCC algorithm [15] uses Total Processor Cycle Consumption (TPCC) mechanism for each task. Min-min [16] is a static algorithm, which identifies a task with minimum execution time and schedules in a machine that could compute the task at minimum completion time compared to other processors. Earliest time to complete matrix [11] ETC [*j*, *m*] gives the completion of task *j* in machine *m*. MCT [17] accumulates the tasks in the task queue and carefully checks each machine to get the minimum completion time. Min-min considers all unmapped task where MCT considers only one task at each scheduling event. ATCS-MCT algorithm is proposed [18], which is a combination of Apparent Tardiness and Cost Setups [19] and MCT algorithm. Max-min [20] is similar to Min-min: Max-min collects the set of incoming task and finds out the minimum completion for each task on each machine. Hence, the machine which gives maximum completion time is chosen to execute the particular task.

The Fair Scheduling [21] algorithm consists of three models, that is, Simple Fair Task Order (SFTO), Adjusted Fair Task Order (AFTO), and Max-Min Fair Share (MMFS). Backfilling [22] is a queue based scheduling technique which makes better utilization of resources and minimizes makespan by allowing task to run out of order. Today, with many flavours of backfilling algorithms, the conservative backfilling [23] and EASY backfilling [24] are the two common variants in traditional backfilling. In conservative backfilling, every task is given a specific reservation when it enters the system. A smaller task is moved forward in the queue as long as it does not delay the previous task. In aggressive backfilling, only the task at the head of the queue has a reservation. A small task is allowed to leap forward as long as it does not delay the task at the head of the queue.

In [25] EG-EDF rule is proposed, which builds the schedule for all tasks incrementally by applying a technique which fills earliest existing gaps in the schedule with newly arriving tasks. If no space is available for the incoming task, EG-EDF rule uses Earliest Deadline First (EDF) strategy for including new task into the existing schedule. In [26] MTEDF which is a combination of Minimum Tardiness Earliest Deadline First (MTEDF) serves as an initial solution to Tabu Search optimization, which is applied in the later stage of the scheduling process. A Genetic Algorithm based workflow optimization model is presented in [27]. The time and cost are two fitness factors considered for optimization. User demand aware scheduling model with hierarchical load balancing narrated in [28] aims to reduce the response time of the job and to improve the resource utilization.

## 3. Hybrid Scheduling Model for Independent Grid Tasks

There are many indexing rules such as FCFS, SJF, EDF, and LJF; these algorithms consider either incoming order or process time or deadline of the task. None of the above-mentioned indexing rules considers the factors together. The ATC rule contemplates the deadline, process time, and current time and with this information the remaining time left to complete the task (deadline) is calculated. If the deadline cannot meet the time constraints then the task is indexed according to the weight. The best gap search is similar to the backfilling policy, where the idle CPU cycles in the existing machine are scheduled, identified, and utilized. This best gap search is applied in the local queue which is the machine queue. Best gap search recognizes the gaps in the machines schedule after an appropriate machine execution is identified for the particular task in the global queue. The results have been compared with various benchmark algorithms. Experimental results show that the proposed algorithm outperforms the benchmark algorithms.

In this work, an independent and nonpreemptive grid task scheduling is initiated. The grid computing environment is realized with “*m*” clusters, each consists of “*n*” processing elements. The scheduling environment is modeled using the [29] notations as *α*∣*β*∣*γ*:

(1) $$\begin{array}{c} R\;|\;r_{i}d_{i}\;|\;\sum W_{i}T_{i}. \end{array}$$

**Figure 1:**
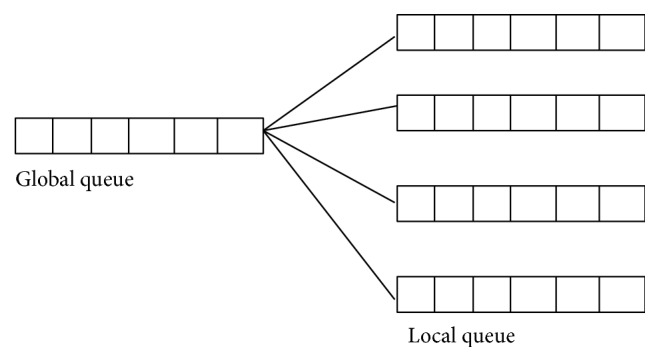
Proposed scheduling system.

The grid environment consists of an unrelated processing element, which is modeled in the first part of a triplet. The parameter specification is shown in Abbreviations section. The proposed work is imposed on arrival time and due, which is represented in the second part. *r*_*i*_ denotes the arrival time of task *i* and *d*_*i*_ denotes due date for task *i*. The objective function minimization of Weighted Tardiness is symbolized in the later part. *W*_*i*_ and *T*_*i*_ denote weight imposed and tardiness on task *i*, respectively. Let “*j*” be a task to be scheduled and “*S*” be a schedule. The incoming tasks are collected in a global queue *U* where a queue deposits all unscheduled tasks. The independent grid task and a non-preemptive scheduling environment are considered in the proposed scheme.  Figure 1 shows the proposed scheduling system. The global queue is a grid level queue, which collects all incoming tasks and local queues are local scheduler queues which are generally cluster queues.

The proposed algorithm has two parts or two phases. On the first phase a composite dispatching rule called ATC is applied [21]. The ATC algorithm is a composition of weighted shortest processing time (WSPT) and minimum slack (MS) rules. The minimum slack of the task “*i*” is calculated using (2). Minimum slack of task *i* denotes the time left for *i* to complete within its deadline:

(2) MSi=Max⁡di−Pi−t,0,

(3) WSPTi=wiPi.

Equation (3) calculates the weighted shortest processing time of the task. Equation (4) is an index function on time *t*. The index value is calculated whenever a new task arrives:

(4) $$\begin{array}{c} I\left(\;t\;\right)=\frac{W_{i}}{P_{i}}*\exp\left(\;\frac{-\max\left(d_{i}-P_{i}-t,0\right)}{K_{1}*P_{\text{avg}}}\;\right). \end{array}$$

The due time range factor *R* and due date tightness factor (tow) decide the value of look ahead parameter. The lower value of *R* denotes that due dates are widely spread out, and higher value of *R* alarms the narrow due dates. In order to accommodate (5) to the grid environment *d*_min⁡_ has been considered as process time of task *i*. *d*_max⁡_ is the actual deadline given to a task. *C*_max⁡_ is a completion time which is the sum of process time calculated from (6). The scheduling algorithm is tabulated in Algorithm 1. Where phase I is the application of ATC algorithm at global queue and phase II is application of BG algorithm at resource level queue:

(5) R=dmax⁡−dmin⁡Cmax⁡,

(6) Cmax⁡=∑j=1nPi.

The look ahead parameter *K*_1_ is computed based on the due date range factor using recommendation given in

(7) K1=4.5+R,R≤0.5,K1=6−2R,R≥0.5.

In the second phase, the best gap [25] has been applied. The best gap search involves searching for a suitable gap in the schedule. The term gap stands for the idle CPU cycle at every schedule. The gap in schedule is found when the numbers of available processing elements are either higher or lower than the requested processing elements of a task. When the numbers of processing elements are more than the required number, the situation leads to a similar situation of internal fragmentation in memory allocation. The best gap search returns number of idle CPU cycles for scheduling the current task *J*. Let *N* be the number of idle CPU cycles. Actually, this situation has three cases to be considered. When a task has no suitable gap in the suitable resource, it has to be pushed in the index order. In the second case, when the task has only one gap it has no other choice and, hence, it should be placed in that hole. Furthermore, when there is more than one gap for a task, the objective function total weighted tardiness is calculated using (8) and then that particular task is being placed in that gap. The schedule, whose total weighted tardiness is low, is preferred:

(8) $$\begin{array}{c} T_{\text{new}}=\overset{n}{\underset{i=0}{\sum }}W_{i}T_{i}. \end{array}$$

## 4. Results and Discussions

The algorithm BG_ATC experimentation is carried out using Intel core i3 processor, with 160 GB HDD and 2 GB RAM. The renowned simulators Gridsim 5.1 [30] is used for realizing the grid environment. Usage of proper workload or a set of data for any simulation is more important in any research [31]. The workload used in this research work is a standard workload format (swf) taken from parallel workload archives. The metacentrum and blue workload traces were used for realizing the grid environment. The SDLC blue job trace is shared by HPC systems group of the San Diego Supercomputer Center (SDSC), which is the leading-edge site of the National Partnership for Advanced Computational Infrastructure (NPACI). There are some random failures imposed on cluster dynamically to realize the real grid computing time. The metacentrum workload traces supplied with such failures. On blue workload traces we artificially imposed the failure. A random machine was chosen to be muted (removed as failure) for random time quantum (in seconds). So this realistic environment cannot be expected to behave in linear fashion all the time. In this study, the concentration was mainly on an independent grid task, to be scheduled under a non-preemptive scheduling, so that no assumptions about the subtasks have been made.

**Figure 2:**
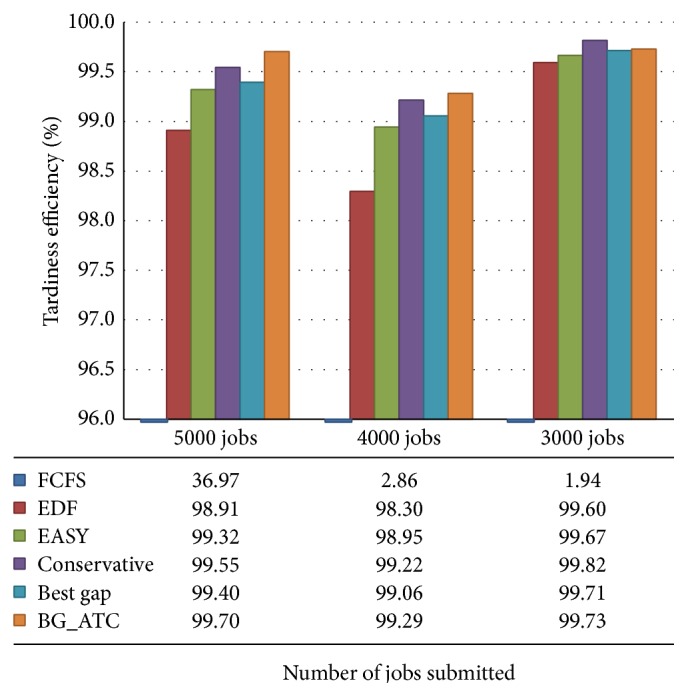
Tardiness efficiency under metacentrum workload.

Figure 2 illustrates the efficiency of various algorithms in minimizing the tardiness under metacentrum workload. There were 3000, 4000, and 5000 jobs submitted to simulation environment. Performance of BG_ATC against various benchmark algorithms is demonstrated. The efficiency is scaled at *y*-axis, where number of jobs submitted are scaled on *x*-axis. The chart tabulates the efficiency of algorithm in percentage. Efficiency of FCFS worst case scenarios is considered. The EDF, EASY backfilling, conservative backfilling, and best gap algorithms performances are nearly equal. The conservative backfilling and BG_ATC are the competitors. The performance of conservative backfilling is higher at low workload situations, whereas on high workload scenarios conservative backfilling was unable to perform because of reservation overhead. The performance of algorithm under grid environment also depends on the resource availability and type of job requirements. Getting suitable resources to execute the jobs may delay the job execution. There is a setback noted in the middle part of the graph which is due to the dynamic job requirements and machine failures.

The conservative backfilling algorithm works well under low CPU loads because it makes reservation for all the jobs available in the queue. Conversely when the incoming queue length is increased, that is, when more jobs are submitted, the conservative backfilling takes more time to schedule the jobs as it attempts to create reservations for all the jobs in queue. This leads to increase in tardiness, wait time, and response time. On the other hand, it gives guaranteed start time for the jobs. But the proposed BG_ATC algorithm performs better under high CPU loads. The backfilling algorithms have to work to fill the gaps in the existing schedule.

**Figure 3:**
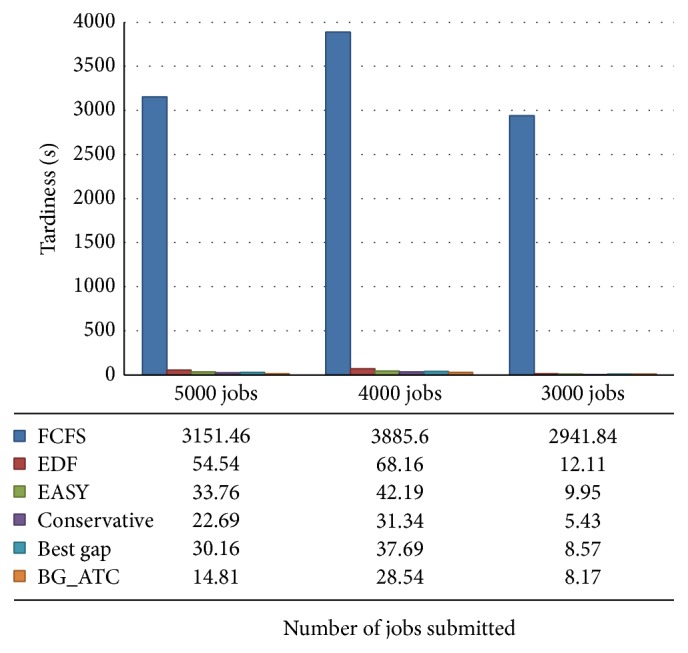
Tardiness analysis on metacentrum workload.

The comparison chart is outlined in Figure 3. Tardiness incurred in seconds under the metacentrum workload. The FCFS and EDF algorithms results in high tardiness, whereas conservative backfilling, EASY backfilling, best gap, and BG_ATC algorithms give very minimum tardiness wherein their witness is small in the graph. The BG_ATC performs well under high and average workloads. Here, surprisingly, conservative backfilling works better under low CPU load on this trace. But, unfortunately, the conservative backfilling algorithms could not perform well under the high CPU load, where the grid environment is generally expected to have high and average CPU loads.

**Figure 4:**
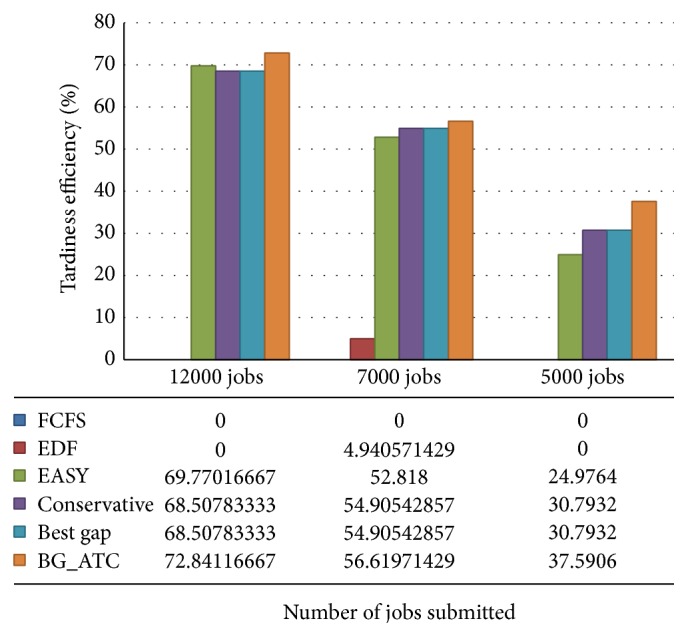
Tardiness efficiency under blue workload.

Efficiency of algorithms in minimizing the tardiness is showcased in Figure 4. The efficiency is scaled on *y*-axis and number of jobs submitted to the environment are scaled on *x*-axis. The First Come First Server and earliest deadline first algorithms perform the worst, so their traces are nullified in the chart. These algorithms consume huge amount of time to complete the tasks. The BG_ATC records a consistent performance on the blue workload. The conservative backfilling and best gap heuristics perform in the same manner. The conservative backfilling allocates the schedule space for all the jobs enter into the queue in FIFO fashion. The best gap also does the same but keeps the jobs in best suitable holes. In memory allocation policy there are some situations where best fit and first fit allocations remain the same. It is observed that the conservative backfilling and best gap search performs identically, where their efficiency remains same in all cases of job submissions. It imitates the situation of memory allocation that is being same on the best fit and first fit circumstances.

**Figure 5:**
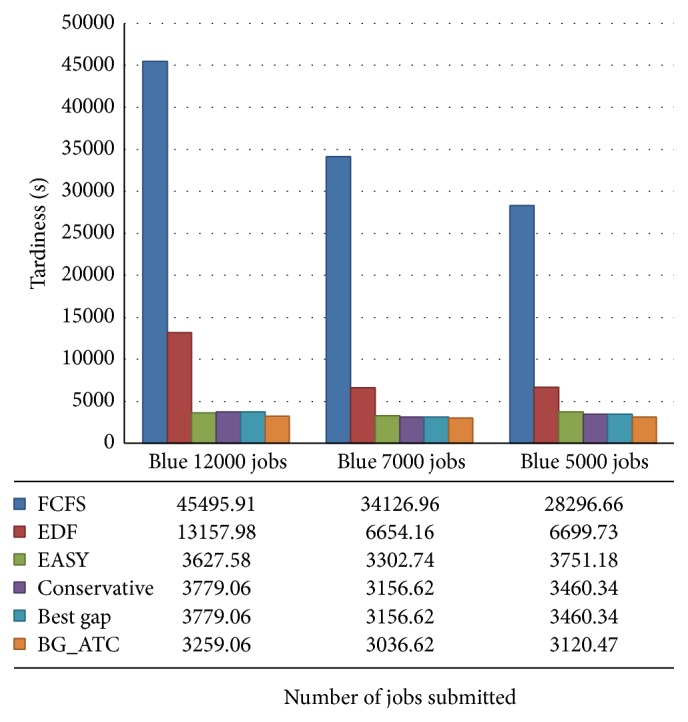
Tardiness analysis on blue workload.

**Figure 6:**
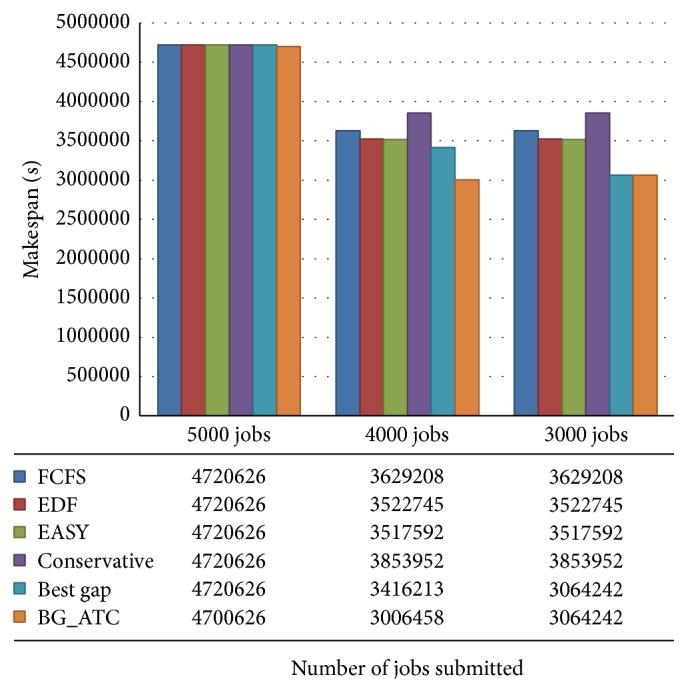
Makespan comparison on metacentrum workload.

The tardiness incurred by the schedule of various algorithms is plotted in Figure 5. The time incurred by FCFS and EDF are too high compared to other algorithms considered in this work. The average makespan of the algorithms has been depicted in Figure 6. The lower value of makespan is the substantiation for higher machine utilization. The makespan of FCFS, EDF, and EASY backfilling algorithms was almost the same. The BG_ATC climbs lower makespan in the chart which is the evidence for the better machine. Since the makespan is the completion time of the last job, makespans of few algorithms are near to one another.

**Figure 7:**
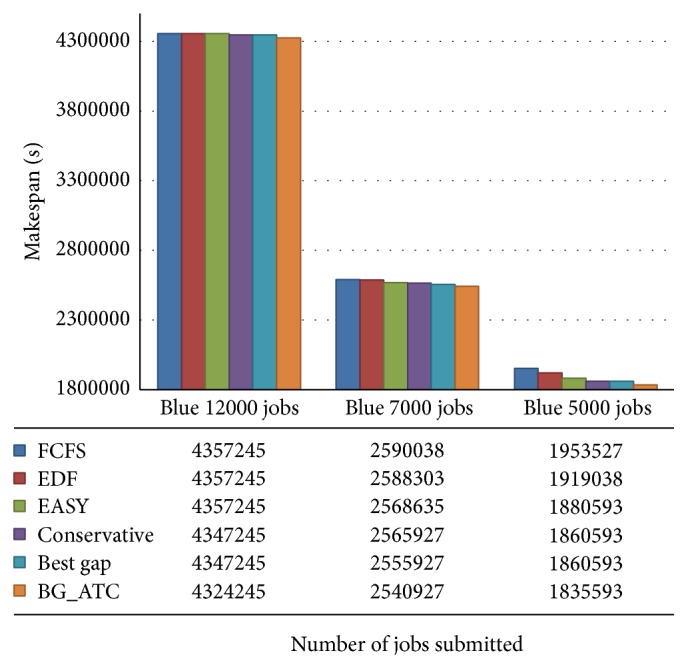
Makespan comparison on blue workload.

Figure 7 demonstrates the comparison of makespan on blue workload. It is observed that the nature of jobs submitted from blue workload traces leads to the near identical makespan. The analysis and study reveal that machine utilization can be identical while the other factors such as tardiness and wait time need not necessarily be identical. The makespan of BG_ATC is comparativetly lower than other algorithms showcasing the efficiency of the proposed algorithm.

**Figure 8:**
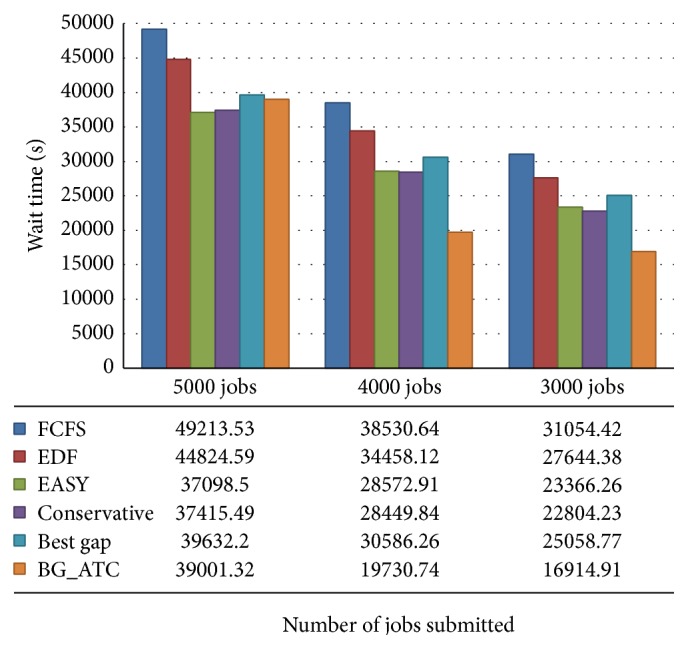
Wait time examination on metacentrum workload.

Figure 8 illustrates the average wait time comparison on metacentrum workload. The proposed scheme BG_ATC outperforms existing method and produces less wait time on lower workloads. When 5000 jobs were submitted, the proposed algorithm incurs higher wait time compared to other algorithms. The type of resource demanded, status of the resource, processing time, and dependence wait conditions are few factor which increase the wait time of the task. The FCFS incurs higher wait time compared to other algorithms. The first come first serve policy concentrates only on arrival time and it does not consider any other measures. The easy and conservative backfilling incurs less wait time when the load is increased. These policies allow the jobs to run out of order. Hence, the waiting time of these two algorithms is lesser.

**Figure 9:**
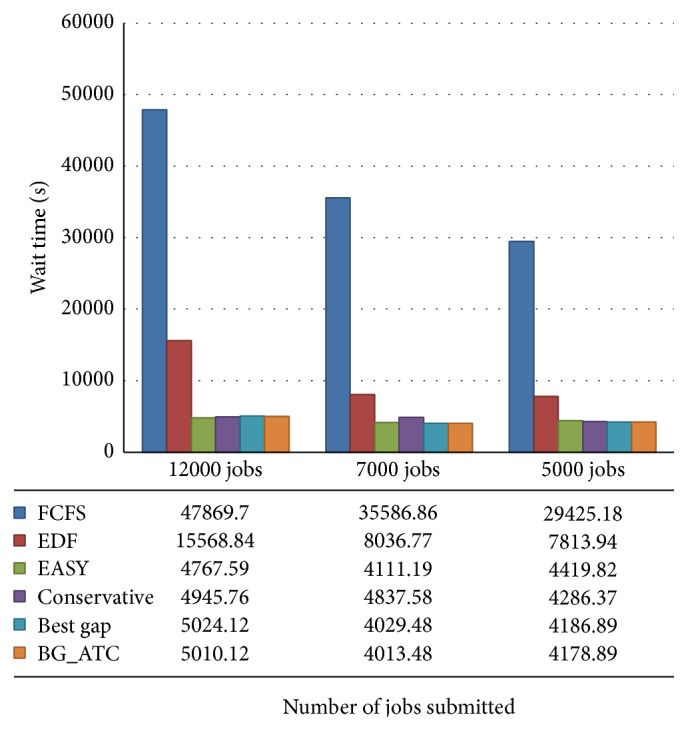
Wait time examination on blue workload.

Figure 9 shows the average wait time on blue workload traces. The best gap heuristics and BG_ATC perform equally under this workload. There is a stable wait time recorded in this trace with all algorithms except FCFS and EDF. It is noted that there is a decrease in wait time recorded for EASY backfilling. The conservative backfilling incurs little higher wait time compared with EASY backfilling. In addition to that, it is observed that conservative backfilling performs slightly better than existing method, but in wait time analysis we observed that EASY backfilling performs better comparatively.

**Figure 10:**
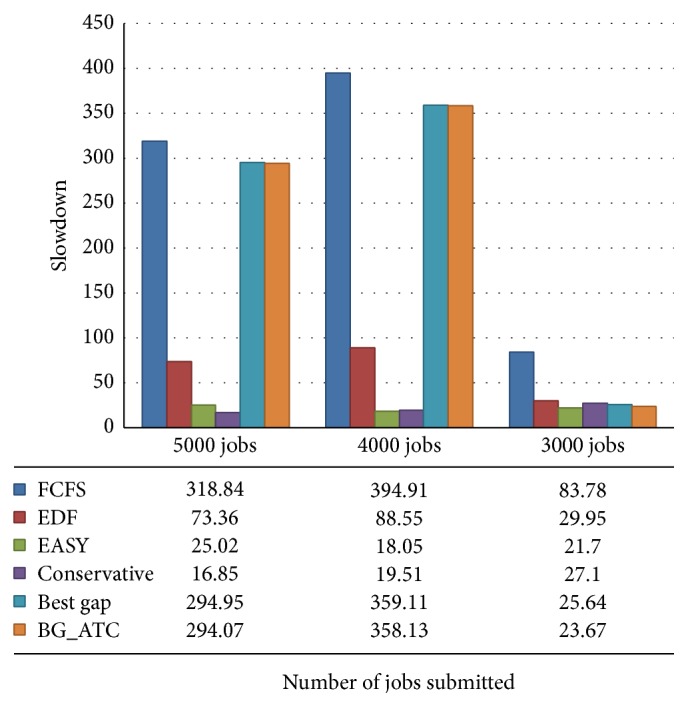
Slowdown comparisons on metacentrum workload.

The slowdown scenario of the metacentrum workload is depicted in Figure 10. The slowdown factor is a normalized frequency which determines the process speed at run time. The slowdown defines the amount of performance degradation due to other applications sharing the cluster. The BG_ATC algorithm reduces the slowdown of cluster 1.08 times compared with the base algorithm best gap. The slowdown performance of EASY backfilling is high with BG_ATC performance. The best gap heuristics and BG_ATC have limitation in slowdown performance. The backfilling variants allow the jobs to run out of order as long as they does not delay the job at the head of the queue. The BG_ATC allow the job wherever it finds the suitable hole. This is the major limitation of using this algorithm.

**Figure 11:**
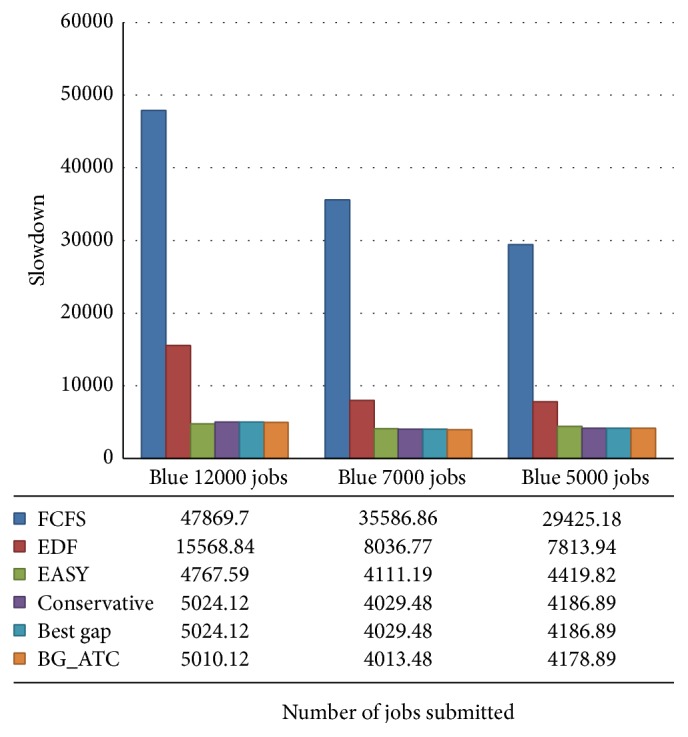
Slowdown comparisons on blue workload.

**Figure 12:**
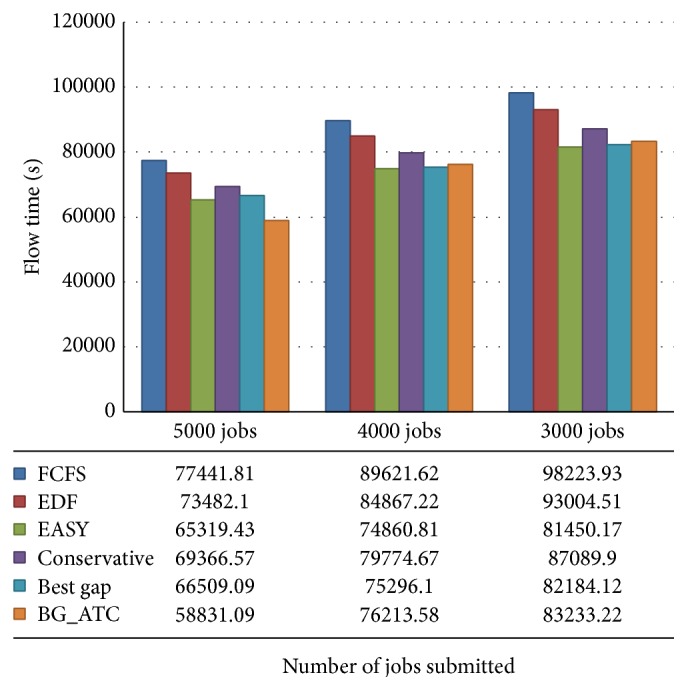
Flow time performance analysis on metacentrum workload.

Figure 11 illustrates the slowdown performance on blue workload. Here it is noted that slowdown of BG_ATC is better than best gap. The blue workload trace has highly heterogeneous job collection which results in completely different performance compared to other workloads.  Figure 12 shows the flow time comparison on metacentrum workload traces. It is noted that, on higher CPU workload, BG_ATC performs better than the lower CPU loads. On submitting 5000 tasks, BG_ATC outperforms other competing algorithms, while with 4000 tasks its performance is trivially higher than the best gap search and considerably higher than EASY backfilling algorithm. In 3000-task submission scenario, around 10% performance reduction is exhibited when compared with best gap search and EASY backfilling.

**Figure 13:**
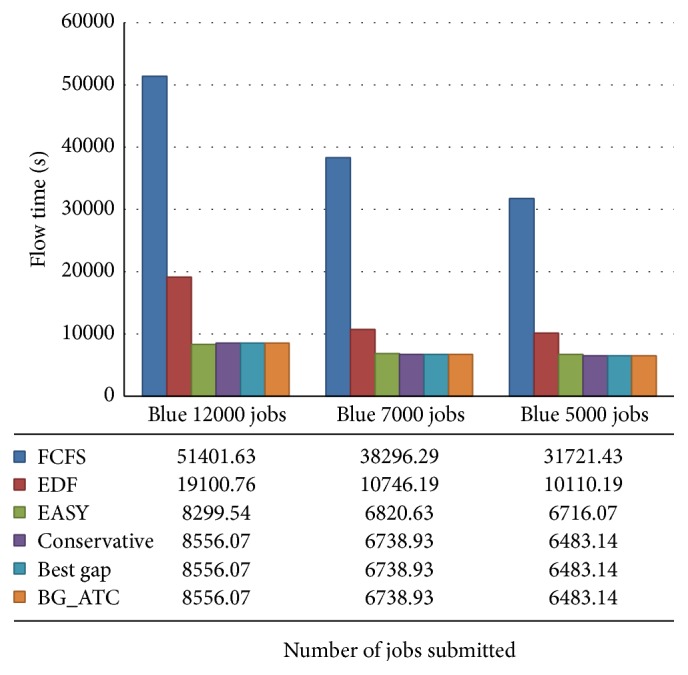
Flow time performance analysis on blue workload.

Figure 13 demonstrates the flow time comparison of various algorithms on blue workload traces. The flow time of EASY backfilling is lower when compared to all other algorithms under high CPU loads. While submitting 7000 and 5000 tasks, the conservative backfilling, best gap, and BG_ATC are performing well and they are equal in responsiveness. Under this scenario, EASY backfilling could not perform better.

## 5. Conclusion

The proposed algorithm includes the composite dispatching rule ATC and heuristic search best gap search. Each incoming task is indexed according to the ATC ranking index at grid level queue. The rank of the task is determined according to the time remained to meet the deadline. Best gap search is applied at the resource level. This search uses the gaps in the existing schedule which increases the resource utilization. Standard workload traces such as metacentrum and blue workloads are used for experiments. These traces were collected from publically available source and have been widely used by the grid community. Experimental result shows that the algorithm outperforms the various benchmark algorithms for heterogeneous environment. Our research work primarily concentrates on reducing the average tardiness and the results were obtained. Our second objective is reduced makespan; we have achieved minimal improvement on efficiently utilizing the machine compared to variants of backfilling policies and best gap heuristics. In this work, we used best gap at resource level so there is only minor improvisation in the makespan compared with close competitive algorithms. The local search algorithms can be incorporated for better performance. These concepts can be adopted in decentralized grid architecture, as the single point of failure is the highly sensitive issue in the centralized architecture. The makespan is another key issue in any scheduling scenario, so that it needs to be given little more importance.

## Figures and Tables

**Algorithm 1:**
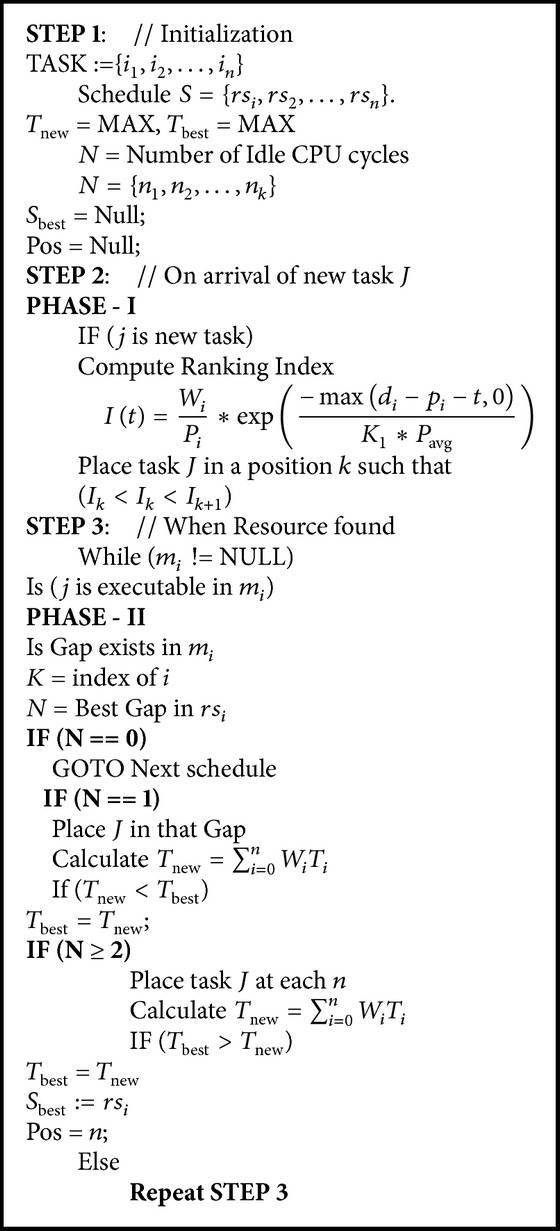
BG_ATC algorithm (BG_ATC).

## Abbreviations

### Parameter Specification

MS(*i*): Minimum slack time for *i*th task
*d*_*i*_: Due date
*P*_*i*_: Process time
*t*: Current time
*W*_*i*_: Weight of the task
*T*_*i*_: Tardiness
WSPT_*i*_: Weighted shortest processing time
*I*(*t*): Ranking index with respect to time
*P*_avg_: Average processing time
*K*_1_: Look ahead parameter
*C*_max⁡_: Completion time
*d*_max⁡_: Maximum deadline of jobs available at incoming queue
*d*_min⁡_: Actual deadline of the task
*R*: Due date range factor
*T*_new_: Victim of minimum weighted tardiness.
